# Heterologous Hyperimmune Polyclonal Antibodies Against SARS-CoV-2: A Broad Coverage, Affordable, and Scalable Potential Immunotherapy for COVID-19

**DOI:** 10.3389/fmed.2021.743325

**Published:** 2021-09-06

**Authors:** Alberto Alape-Girón, Andrés Moreira-Soto, Mauricio Arguedas, Hebleen Brenes, Willem Buján, Eugenia Corrales-Aguilar, Cecilia Díaz, Ann Echeverri, Marietta Flores-Díaz, Aarón Gómez, Andrés Hernández, María Herrera, Guillermo León, Román Macaya, José Arturo Molina-Mora, Javier Mora, Aarthi Narayanan, Alfredo Sanabria, Andrés Sánchez, Laura Sánchez, Álvaro Segura, Eduardo Segura, Daniela Solano, Claudio Soto, Jennifer L. Stynoski, Mariángela Vargas, Mauren Villalta, Jan Felix Drexler, José María Gutiérrez

**Affiliations:** ^1^Instituto Clodomiro Picado, School of Microbiology, University of Costa Rica, San Pedro, Costa Rica; ^2^School of Medicine University of Costa Rica, San Pedro, Costa Rica; ^3^Institute of Virology, Charité Medical University of Berlin, Berlin, Germany; ^4^Instituto Costarricense de Investigación y Enseñanza en Nutrición y Salud, Ministry of Health, Cartago, Costa Rica; ^5^Caja Costarricense del Seguro Social, San Jose, Costa Rica; ^6^Research Center for Tropical Diseases, School of Microbiology, University of Costa Rica, San Pedro, Costa Rica; ^7^National Center for Biodefense and Infectious Diseases, College of Science, George Mason University, Fairfax, VA, United States

**Keywords:** heterologous antibodies, passive immunotherapy, COVID-19, SARS-CoV-2, hyperimmune polyclonal antibodies, convalescent plasma, monoclonal antibodies

## Introduction

The emergence and dissemination of the severe acute respiratory syndrome coronavirus 2 (SARS-CoV-2) and the resulting COVID-19 pandemic triggered a global public health crisis. Although several SARS-CoV-2 vaccines have been developed, demand far exceeds supply, access to them is inequitable, and thus, populations in low- and middle-income countries are unlikely to be protected soon (1). Furthermore, there are no specific therapies available, which is a challenge for COVID-19 patient care (2). Thus, the appearance of SARS-CoV-2 variants and reports of reinfections associated with immune escape (3, 4) highlight the urgent need for effective and broad coverage COVID-19 therapeutics.

Intravenous administration of human or heterologous antibodies is a therapy successfully used in patients with viral respiratory diseases (5). Accordingly, formulations containing SARS-CoV-2 specific antibodies are an attractive therapeutic option for COVID-19 patients (6). SARS-CoV-2 specific antibodies could limit infection by direct virion neutralization and/or by targeting infected cells for elimination via complement or antibody-mediated cytotoxicity (6).

Specific SARS-CoV-2 antibody-based therapeutics include convalescent plasma (CP), monoclonal antibodies (mAbs), human polyclonal IgG formulations purified from CP or transgenic animals, and heterologous hyperimmune polyclonal antibodies (pAbs) (6). Although the window for using antibody-based therapeutics varies, clinical data show that they are mainly effective if administered early after symptoms onset (6).

## Convalescent Plasma Transfusion

CP transfusion contributes to viral clearance and improves patient survival when administered promptly and has been therapeutically used worldwide during the COVID-19 pandemic in hospitalized patients (6, 7). However, it is worth mentioning that in some developing countries CP transfusion may heighten the risk of transmission of blood-borne pathogens if proper donors screening and virus testing procedures are not applied (8, 9). Furthermore, uncertainty remains about the therapeutic efficacy of CP transfusion, as controlled clinical trials have provided variable results in terms of mortality and need for mechanical ventilation (7). The diversity among reported results is likely due to the heterogeneity of trial designs, anti-SARS-CoV-2 plasma titer variation, and differences in latency to product administration (7). Therefore, more studies are required to establish the optimal doses and timing for CP transfusion to COVID-19 patients.

## Monoclonal Antibodies

Over 80 mAbs have been shown to block the interaction between the SARS-CoV-2 S1 glycoprotein and its cellular receptor, thus neutralizing virus infectivity *in vitro* (10). Some of those mAbs demonstrate therapeutic efficacy to curtail viral burden and lung inflammation in animal models (10). The neutralization mechanisms of mAbs against SARS-CoV-2 *in vivo* are not fully understood, but optimal protection correlates with Fc effector functions (11).

Approximately 30 SARS-CoV-2 neutralizing mAbs are undergoing clinical trials in COVID-19 patients (10). Some were granted emergency authorization since they reduced viral load, disease severity, and hospitalization in randomized, controlled phase II clinical trials (10). However, mAbs are unaffordable for healthcare systems in many developing countries due to their high cost (> USD 1,500/vial), meaning that most infected people would not have access to them (12).

Another obstacle for COVID-19 therapy with mAbs is the emergence of viral variants harboring changes in the receptor-binding domain (RBD) of the S1 glycoprotein (13). The variants of concern (VoC) exhibit enhanced transmissibility or virulence, circulate worldwide, and include those designated as alpha, beta, epsilon, gamma, and delta, first detected in the UK, South Africa, Brazil, USA, and India, respectively (13). Therapeutic mAbs, and antibodies in the plasma of vaccinated or convalescent individuals, fail to neutralize VoC efficiently (13–17).

## Human and Heterologous Hyperimmune Polyclonal Antibody Formulations

Intravenous IgG (IVIGs) formulations purified from CP pools are a cheaper option (> USD 300/vial) than mAbs for therapy of COVID-19 patients (18, 19), which furthermore could be used prophylactically. Human IVIGs could be also purified from the plasma of genetically modified transchromosomic bovines hyperimmunized with SARS-CoV-2 antigens (20).

Preparation of IVIGs formulations from CP is feasible for some developing countries. However, it requires strict donor screening for high levels of SARS-CoV-2 neutralizing antibodies, as well as the absence of blood-borne pathogens and antibodies against human leucocyte or neutrophil antigens to limit the risks of Transfusion Related Acute Lung Injury (21). Thus, this therapy depends on rigorous blood bank systems that are often scarce in low- and middle-income countries (22). Due to these logistical hurdles and lack of infrastructure, it is difficult for most developing countries to rapidly establish large-scale manufacturing capacity to prepare IVIGs formulations against SARS-CoV-2 (23).

Results were recently reported about a single-center, single-blind, placebo-controlled phase I/II clinical trial (NCT04521309) with anti-SARS-CoV-2 IVIGs purified from CP in Pakistan with 55 hospitalized severe or critical COVID-19 patients (24). The data showed that this immunotherapy is safe, increases survival, and reduces the disease progression risk (24). Similarly, IVIGs purified from the plasma of transchromosomic bovines hyperimmunized with the SARS-CoV-2 S1 glycoprotein are well-tolerated by non-hospitalized COVID-19 patients according to a phase Ib clinical trial (NCT0446917) performed in the USA (20). This pAbs formulation will be evaluated in a phase II/III clinical trial (NCT04518410) in non-hospitalized COVID-19 patients ongoing in the USA.

Another promising therapy for COVID-19 patients is the intravenous administration of heterologous pAbs, purified from plasma of animals hyperimmunized with SARS-CoV-2 proteins (23). Such formulations of intact or fragmented hyperimmune equine/ovine pAbs are therapeutics with a proven path to regulatory approval, which have been successfully used worldwide for decades as therapies against rabies virus infection or as antivenoms to treat patients bitten or stung by venomous animals (25). Several manufacturers from developed and developing countries regularly supply formulations of heterologous pAbs as therapeutic antivenoms which comply with Good Manufacturing Practices (GMPs) and show good safety and efficacy profiles in the treatment of envenomings by animal bites and stings (26–29). The early and late adverse reactions induced by GMP-prepared formulations of heterologous pAbs are mainly mild, and if occur can be monitored and pharmacologically controlled (30, 31). Indeed, heterologous pAbs formulations are approved by the Food and Drug Administration, as therapies for envenoming by snakes, scorpions, and spiders, as well as for digoxin poisoning, botulism, and as anti-thymocyte globulin in immunosuppressive regimens (32). They are also approved by the European Medicines Agency as therapies for avian influenza and rabies virus infections, snakebite envenoming, hemolytic uremic syndrome, and colchicine poisoning.

Formulations of equine pAbs against the SARS-CoV S1 glycoprotein control coronavirus infectivity in cultured cells and animal models (33). Similarly, IgG and F(ab')2 formulations from horses immunized with MERS-CoV virus-like particles exhibit *in vivo* neutralization (33). Recently, three F(ab')2 formulations from plasma of horses hyperimmunized with recombinant SARS-CoV-2 S1 glycoprotein or its RBD were described (34–37). These formulations are significantly more potent than COVID-19 convalescent plasma at neutralizing SARS-CoV-2 infectivity (35–37). Moreover, a multi-center, double-blind, placebo-controlled phase II/III clinical trial (NCT04494984) with RBD-specific equine F(ab')2 fragments performed in Argentina showed that this immunotherapy is well-tolerated and that clinical improvement of hospitalized severe COVID-19 patients is achieved (38). Five clinical trials are registered to test equine F(ab')2 formulations against SARS-CoV-2 that were produced by anti-venom-manufacturing laboratories in India, Argentina, Brazil (two trials), and Mexico (NCT04834908, NTC04913779, NCT04834089, NCT04573855, NCT04514302), although patient recruitment has only begun in India and Argentina.

In contrast to mAbs, equine pAbs against the SARS-CoV-2 S1 glycoprotein recognize multiple epitopes, which reduces the risk of viral escape. Long-term experience underscores that equine pAbs can be produced on a large scale and at a much lower cost (< USD 35/vial) than mAbs or IVIGs. The high neutralization potency of equine pAbs (35–37), likely results from a higher affinity than pAbs from CP because horses are exposed to optimized hyperimmunization protocols using repeated doses of viral antigens in the presence of adjuvants. Also, it is worth mentioning that equine antibodies have a low risk of viral contamination, considering that the horse plasma fractionation technologies include viral removal/inactivation steps (39).

## The Costa Rican Experience

**Figure 1:**
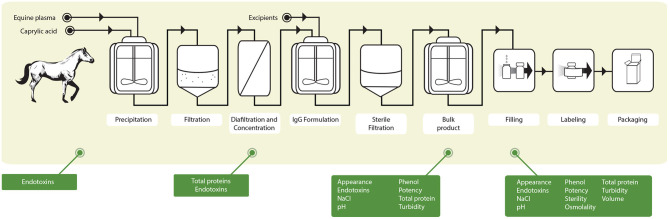
Schematic summary of the protocol followed for the production of the anti-SARS-CoV-2 ICP at the Clodomiro Picado Institute in Costa Rica. Groups of horses were immunized with either the S1 viral protein or a mixture of S1, N and SEM viral proteins. After immunization, horses were bled, and the plasma was separated and fractionated according to the scheme shown. The green boxes depict the quality control assays carried out during the process of fractionation and of the final product. In addition, ELISA antibody titers against viral proteins and neutralization of virus infectivity in cell culture were done by plaque reduction neutralization tests to ensure viral neutralization efficacy [see Leon et al. (37) for details].

Given the urgent need for affordable COVID-19 treatments, two intact-IgG formulations were produced at the Clodomiro Picado Institute (ICP) of the University of Costa Rica from the plasma of horses hyperimmunized with either S1 (anti-S1) or a mixture of S1, N, and SEM mosaic (anti-Mix) SARS-CoV-2 recombinant proteins (37) (Figure 1). Pools of 25 L of hyperimmune plasma were used to prepare pilot batches of 500 vials of 10 mL, which were manufactured in 70 days, from the start of horse immunization to the final quality control tests of the products (37) (Figure 1). The fractionation process included two steps with robust viral inactivation/removal activity (caprylic acid precipitation and phenol addition) to ensure the viral safety of the products, as required by the WHO (21). The capacity of both formulations to neutralize virus infectivity (USA-WA1/2020 isolate) *in vitro* is 80 times higher than that of pooled human convalescent plasma (37). Additionally, the equine IgGs in these formulations activate human FcγRIIIA *in vitro*, suggesting downstream mediation of effector functions via interaction with Fc receptors in immune cells (37).

We compared the clinical safety and efficacy of the anti-S1 and anti-Mix pAbs formulations in a Bayesian pick-the-winner type phase IIa clinical trial (NCT04610502) in 27 COVID-19 patients with two or more risk factors. The single-dose immunotherapy with 550 mg of equine pAbs was well-tolerated, as adverse reactions were mild and resolved without sequelae. Most adverse reactions were cutaneous and easily controlled with a standard regimen of antihistamine drugs and steroids, as those induced by horse-derived antivenoms produced at the ICP and used in the therapy of snakebite envenomings (26, 27). Moreover, a decrease in viral load following infusion of these formulations and the good clinical recovery of most patients provide preliminary support for the clinical rationale of this COVID-19 therapy. A randomized, multi-center, double-blind, placebo-controlled, dose-finding phase IIb/III clinical trial (NCT04838821) with 173 patients is underway at hospitals of the Costa Rican Social Security Fund (Caja Costarricense del Seguro Social) to conclusively establish safety and efficacy, and to determine the optimal dose of the anti-S1 equine pAbs formulation (designated “Anti-SARS-CoV-2 ICP”) to treat moderate and severe COVID-19 cases. If the ongoing trial demonstrates the efficacy and safety of this formulation, its clinical use would be therapeutic and not prophylactic, owing to the heterologous nature of equine antibodies.

Both formulations of equine pAbs prepared at the ICP effectively inhibit the infectivity of two SARS-CoV-2 early isolates (WA1/2020 and Gisaid_EPI_ISL_406862, from USA and Germany, respectively) and five VoCs (alpha, beta, epsilon, gamma, and delta) with similar neutralizing potencies (40). The 50% Inhibitory Concentration (IC50) range in a plaque reduction neutralization assay was 0.146–1.078 μg/mL, which is much lower than the antibody concentrations presumably circulating in the patients' blood in the ongoing phase IIb/III clinical trial, given treatment doses of 12–56 mg/kg.

## Concluding Remarks

The preclinical data, along with those obtained in completed clinical trials, prompt further investigations regarding the potential of equine pAbs targeting SARS-CoV-2 as a broad coverage, low-cost, and scalable treatment for COVID-19. Similar to antivenoms, those therapeutics can be readily produced under GMPs in low- and middle-income countries that have this technology in place and distributed at prices accessible to resource-constrained economies. WHO standardized guidelines are available to produce heterologous pAbs for therapeutic use in humans (21), which should allow for the worldwide production of SARS-CoV-2 specific formulations within a relatively short time. Following the protocol described in Leon et al. (37), after immunizing 20 horses with S1 protein produced in insect cells, industrial batches of 5,000 vials of 10 mL of Anti-SARS-CoV-2 ICP can be manufactured from 250 L of hyperimmune plasma pools every 2 months. Antivenom-manufacturing laboratories operating in Argentina, Australia, Bolivia, Brazil, Colombia, Costa Rica, Egypt, France, Mexico, India, Peru, South Africa, Thailand, UK, USA, and Venezuela could quickly adapt existing platforms to produce equine pAbs against SARS-CoV-2 or future emerging viral agents to cope with the global need for affordable therapeutic options during pandemic crises.

If ongoing clinical trials corroborate the safety and efficacy of equine pAbs against SARS-CoV-2, the international community should support existing antivenom manufacturing laboratories in several countries to allow for the production of these new therapeutic antibody formulations with their platforms. If this is done cooperatively and effectively, the world, and particularly low- and middle-income countries, could have a readily available therapeutic option for COVID-19 patients.

## Author Contributions

MF-D, AA-G, JS, AM-S, and JMG took the lead in writing the manuscript. All authors provided critical feedback, take responsibility for the overall content and integrity of the work, concur with the submission and have contributed to, read, and approved the final version.

## Conflict of Interest

Several authors of this manuscript are employees of the Instituto Clodomiro Picado at the University of Costa Rica, a public research institute with no commercial interests, where these antibody formulations were developed, and where they eventually will be manufactured for use in the Costa Rican public health system.

## Publisher's Note

All claims expressed in this article are solely those of the authors and do not necessarily represent those of their affiliated organizations, or those of the publisher, the editors and the reviewers. Any product that may be evaluated in this article, or claim that may be made by its manufacturer, is not guaranteed or endorsed by the publisher.
